# Cardiovascular Event Predictors in Hospitalized Chronic Kidney Disease (CKD) Patients: A Nationwide Inpatient Sample Analysis

**DOI:** 10.7759/cureus.47912

**Published:** 2023-10-29

**Authors:** Fidelis Uwumiro, Chikodili Nebuwa, Chimaobi O Nwevo, Victory Okpujie, Osasumwen Osemwota, Emeka S Obi, Omamuyovbi Nwoagbe, Ejiroghene Tejere, Joycelyn Adjei-Mensah, Charles T Ogbodo, Christopher N Ekeh

**Affiliations:** 1 Internal Medicine, Our Lady of Apostles Hospital, Akwanga, NGA; 2 Internal Medicine, Nuvance Health Medical Practice, New York, USA; 3 Medicine and Surgery, University of Calabar Teaching Hospital, Calabar, NGA; 4 Internal Medicine, Central Hospital Benin, Benin City, NGA; 5 Internal Medicine, Western Illinois University, Macomb, USA; 6 Healthcare Administration, College of Public Health, East Tennessee State University, Johnson City, USA; 7 Internal Medicine, University of Port Harcourt Teaching Hospital, Port Harcourt, NGA; 8 Internal Medicine, Kharkiv National Medical University, Kharkiv, UKR; 9 Internal Medicine, Achimota Hospital, Achimota, GHA; 10 Internal Medicine, Médecins Sans Frontières, General Hospital Anka, Anka, NGA; 11 Internal Medicine, Surgicare Hospital, Abuja, NGA

**Keywords:** cardiac arrhythmias, acute coronary syndrome, cardiac sudden death, nationwide inpatient sample (nis), chronic kidney disease, cardiovascular events

## Abstract

Introduction: This study seeks to confirm the risk factors linked to cardiovascular (CV) events in chronic kidney disease (CKD), which have been identified as CKD-related. We aim to achieve this using a larger, more diverse, and nationally representative dataset, contrasting with previous research conducted on smaller patient cohorts.

Methods: The study utilized the nationwide inpatient sample database to identify adult hospitalizations for CKD from 2016 to 2020, employing validated ICD-10-CM/PCS codes. A comprehensive literature review was conducted to identify both traditional and CKD-specific risk factors associated with CV events. Risk factors and CV events were defined using a combination of ICD-10-CM/PCS codes and statistical commands. Only risk factors with specific ICD-10 codes and hospitalizations with complete data were included in the study. CV events of interest included cardiac arrhythmias, sudden cardiac death, acute heart failure, and acute coronary syndromes. Univariate and multivariate regression models were employed to evaluate the association between CKD-specific risk factors and CV events while adjusting for the impact of traditional CV risk factors such as old age, hypertension, diabetes, hypercholesterolemia, inactivity, and smoking.

Results: A total of 690,375 hospitalizations for CKD were included in the analysis. The study population was predominantly male (375,564, 54.4%) and mostly hospitalized at urban teaching hospitals (512,258, 74.2%). The mean age of the study population was 61 years (SD 0.1), and 86.7% (598,555) had a Charlson comorbidity index (CCI) of 3 or more. At least one traditional risk factor for CV events was present in 84.1% of all CKD hospitalizations (580,605), while 65.4% (451,505) included at least one CKD-specific risk factor for CV events. The incidence of CV events in the study was as follows: acute coronary syndromes (41,422; 6%), sudden cardiac death (13,807; 2%), heart failure (404,560; 58.6%), and cardiac arrhythmias (124,267; 18%). A total of 91.7% (113,912) of all cardiac arrhythmias were atrial fibrillations. Significant odds of CV events on multivariate analyses included: malnutrition (aOR: 1.09; 95% CI: 1.06-1.13; p<0.001), post-dialytic hypotension (aOR: 1.34; 95% CI: 1.26-1.42; p<0.001), thrombophilia (aOR: 1.46; 95% CI: 1.29-1.65; p<0.001), sleep disorder (aOR: 1.17; 95% CI: 1.09-1.25; p<0.001), and post-renal transplant immunosuppressive therapy (aOR: 1.39; 95% CI: 1.26-1.53; p<0.001).

Conclusion: The study confirmed the predictive reliability of malnutrition, post-dialytic hypotension, thrombophilia, sleep disorders, and post-renal transplant immunosuppressive therapy, highlighting their association with increased risk for CV events in CKD patients. No significant association was observed between uremic syndrome, hyperhomocysteinemia, hyperuricemia, hypertriglyceridemia, leptin levels, carnitine deficiency, anemia, and the odds of experiencing CV events.

## Introduction

Chronic kidney disease (CKD) affects millions of individuals worldwide [1,2]. In addition to impairing renal function, CKD is associated with an elevated risk of cardiovascular (CV) events, such as myocardial infarction, stroke, and heart failure [3-5]. While traditional CV risk factors like hypertension, diabetes, and dyslipidemia are well-studied and widely reported in the general population, CKD-specific risk factors are less studied.

The burden of CKD has been steadily increasing, with a substantial impact on public health [6]. CKD is known to be linked to a significantly higher risk of CV events compared to the general population, and it is more likely to lead to CV disease than progress to end-stage kidney disease requiring renal replacement therapy [7,8]. However, it remains uncertain whether CKD-specific CV risk factors, which have been reported in smaller studies as predictors of vascular pathology and justifying the higher incidence of CV morbidity in CKD patients, hold similar predictive value when applied to a more diverse cohort of CKD patients. Researchers already suspect that existing prognostic models for CV events among individuals with CKD may have limited clinical applicability [9]. Consequently, validating CKD-specific risk factors for CV events using larger and more representative data is a crucial step toward appropriate risk stratification, prevention, and targeted management in this vulnerable population. Previous studies investigating the association between CKD and CV events have identified several predictors of CV events that are thought to be specific to CKD patients. These predictors include uremic syndrome, thrombophilia, sleep disorders, post-kidney transplant immunosuppressive therapy, post-dialytic hypotension, obesity, malnutrition, hyperuricemia, hypertriglyceridemia, hyperhomocysteinemia, carnitine deficiency, and anemia of CKDs [10-13]. However, these studies are limited by small sample sizes, selection bias, and a lack of generalizability due to single-center settings. Consequently, conducting a study using a more representative dataset is warranted.

The primary objective of this study is to examine the predictive value and generalizability of CV risk factors in hospitalized CKD patients reported in the literature, using the National Inpatient Sample (NIS) data. Additionally, we aim to explore sociodemographic attributes and the prevalence of traditional CV risk factors in CKD patients. By examining a large and diverse CKD cohort, we intend to enhance our understanding of the unique risk factors contributing to the incidence of CV events in this population.

## Materials and methods

Study design and data source

This study utilized data from the NIS, a comprehensive repository of all inpatient stays across the United States. The NIS offers a unique opportunity to examine a large cohort of CKD patients and assess the reliability of established and emerging risk factors for CV events.

The NIS is a valuable dataset widely utilized in healthcare research and policy analysis. It is the largest all-payer inpatient care database in the United States, providing comprehensive information on hospital stays across the country. The NIS is managed by the Healthcare Cost and Utilization Project (HCUP), a federal-state-industry partnership sponsored by the Agency for Healthcare Research and Quality (AHRQ). It contains data from over 4,000 hospitals and 7 million hospital stays annually, capturing a representative sample of approximately of U.S. hospitalizations excluding rehabilitation and long-term acute care hospitals. Researchers rely on the NIS to examine trends, outcomes, and healthcare utilization patterns on a national scale. The dataset is weighted to obtain national estimates for the entire US population [14,15].

Study population and variables

The NIS databases spanning the years 2017 to 2020 were queried using the ICD-10-CM/PCS coding system. Within this dataset, all adult hospitalizations for a principal diagnosis of CKD were included for analysis. Traditional risk factors associated with CV events, namely advanced age (60 years or older), a family history of CV disease, physical inactivity or frailty, tobacco smoking, hypertension, diabetes mellitus, and hypercholesterolemia, were identified using the ICD-10-CM/PCS codes [16-18]. CKD-specific risk factors for CV events were identified using the International Classification of Diseases, Tenth Revision, Clinical Modification and Procedure Coding System (ICD-10-CM/PCS) and Clinical Classifications Software Refined (CCSR) codes. ICD-10-CM/PCS and CCSR codes were also utilized to identify the most prevalent CV events observed in individuals with CKD, including acute coronary syndromes, cardiac arrhythmias, sudden cardiac death (SCD), and heart failure, utilizing ICD-10-CM/PCS codes [19]. To evaluate the burden of comorbidities, we employed the Charlson comorbidity index (CCI) score. Specifically, we utilized Sundararajan's adaptation of the modified Deyo's CCI [20], which has been tailored for population-based research. This adaptation categorizes the CCI into four groups based on ascending risk for mortality. A score surpassing 3 signifies an approximate 25% 10-year mortality rate, while scores of 2 or 1 correspond to 10% and 4% 10-year mortality rates, respectively. CKD severity was defined by the presence of codes for end-stage renal disease (ESRD) or the need for renal replacement therapy in hospitalization records.

Outcome measures

We estimated sociodemographic characteristics as well as the frequency of both traditional and CKD-specific risk factors associated with CV events among individuals hospitalized with CKD. Subsequently, we conducted an analysis to ascertain the odds of experiencing CV events associated with each CKD-specific risk factor previously documented in the literature.

Statistical analysis

All data analyses were carried out using Stata® software version 17.0MP (StataCorp LLC, College Station, TX, USA). In order to adhere to the regulations established by the HCUP for the usage of the NIS database, all analyses were conducted and results were reported based on the weighted sample. Prevalence rates were determined by calculating proportions, and multivariate regression models were employed to analyze outcome measures, accounting for potential confounding variables including traditional risk factors for CV disease and other patient and hospital-level variables included in Table 1. A significance threshold of 0.05 was maintained to assess the statistical significance of the obtained outcomes.

Ethical considerations

This study utilized de-identified data from the NIS database. As a limited dataset, NIS adheres to the Health Insurance Portability and Accountability Act (HIPAA) guidelines, with all direct patient and hospital identifiers removed. Consequently, institutional review board review was not sought.

Data availability statement

The NIS is the largest publicly available, all-payer inpatient care database containing data on more than seven million US hospital stays annually. NIS datasets are available through the HCUP central distributor on request.

## Results

Sociodemographic characteristics of the study population

The analysis comprised a total of 690,375 hospitalizations for a primary diagnosis of CKD. The study population exhibited a predominance of male patients (375,564, 54.4%), admitted during weekdays (544,706; 78.9%), and primarily received care at urban teaching hospitals (512,258, 74.2%). The average age of the study population was 61 years (SD 0.1), with 86.7% (598,555) of them having a CCI score of 3 or higher. At least 97.5% of the total CKD hospitalizations were insured under Medicare, Medicaid, or Private including HMO (673,116) compared to self-pay/uninsured (17,259). Table 1 summarizes the sociodemographic characteristics and the prevalence of traditional and CKD-specific risk factors for CV events in the study population.

**Table 1:** Sociodemographic characteristics and prevalence of risk factors for cardiovascular events in the study population SD, standard deviation; US$, United States dollar; CKD, Chronic kidney disease ** Identified from existing literature

Variables	n (%)
Women	314,811 (45.6)
Men	382,467 (54.4)
Mean age (years ± SD)	61.2 ± 0.1
Weekend admission	145,669 (21.1)
Charlson comorbidity index score	
0	276 (0.04)
1	414 (0.06)
2	91,130 (13.2)
≥3	598,555 (86.7)
Insurance type	
Medicare	477,049 (69.1)
Medicaid	104,937 (15.2)
Private	91,130 (13.2)
Uninsured	17,259 (2.5)
Median annual income in patient’s zip code (US$)	
1-43,999	276,840 (40.1)
44,000-55,999	177,426 (25.7)
56,000-73,999	137,384 (19.9)
≥ 74,000	98,724 (14.3)
CKD-specific risk factors**	
Uremic syndrome	138 (0.02)
Thrombophilia	9,665 (1.4)
Sleep disorder	34,518 (5.0)
Post-kidney transplant immunosuppressive therapy	12,427 (1.8)
Post-dialytic hypotension	44,874 (6.5)
Obesity	69,728 (10.1)
Malnutrition	169,832 (24.6)
Hyperuricemia	2,071 (0.3)
Hypertriglyceridemia	2,071 (0.3)
Hyperhomocysteinemia	138 (0.02)
Carnitine deficiency	7 (0.001)
Anemia of chronic kidney disease	382 (55.4)
Traditional cardiovascular risk factors	
Old age	396,966 (57.5)
Inactivity	55 (0.008)
Frailty	4,142 (0.6)
History of tobacco smoking	249,916 (36.2)
Hypercholesterolemia	325,857 (47.2)
Arterial hypertension	15,188 (2.2)
Diabetes mellitus	42,803 (6.2)
Hospital characteristics/location	
Northeast	117,363 (17.0)
Midwest	133,933 (19.4)
South	310,668 (45.0)
West	129,100 (18.7)
Rural	35,900 (5.2)
Urban non-teaching	142,217 (20.6)
Urban teaching hospital	512,285 (74.2)
Race	
White American	272,698 (39.5)
Black	239,560 (34.7)
Hispanic	119,435 (17.3)
Asian or pacific islander	28,995 (4.2)
Native American	6,904 (1.0)
Others	22,782 (3.3)

CV events

At least one CV event was recorded in 436,317 admissions (63.2%) in this study, revealing an incidence rate of 158 adverse CV events per 1000 admissions for CKD in the study period. About 84.1% of all hospitalizations (580,605) had at least one traditional risk factor, while 65.4% (451,505) included at least one CKD-specific risk factor for CV events. The incidences of specific CV events observed were as follows: acute coronary syndromes (6%), cardiac arrhythmias (18%), sudden cardiac death (2%), and heart failure (58.6%). Among cardiac arrhythmias, 91.7% (113,912) were identified as atrial fibrillations (Table 2).

**Table 2:** Prevalence of specific risk factors and association with adverse cardiovascular events on multivariate regression analyses *Significant at p-values <0.05 CKD, chronic kidney disease; CI, confidence interval; aOR, adjusted odds ratio

Risk factor	Prevalence, (n, %)	Multivariate regression analyses, aOR (95% CI; p-value^*^)			
		Heart failure	Acute coronary syndromes	Sudden cardiac death	Cardiac arrhythmias
Uremic syndrome	138 (0.02)	0.57 (0.24-1.34; p=0.196)	1.06 (0.14-8.31; p=0.953)	2.32 (0.17-31.6; p=0.529)	0.51 (0.12-2.22; p=0.375)
Malnutrition	169,832 (24.6)	1.07 (1.04-1.14; p<0.001)	1.05 (0.99-1.11; p=0.118)	1.20 (1.10-1.32; p<0.001)	1.08 (1.04-1.22; p<0.001)
Post-dialytic hypotension	44,184 (6.4)	1.16 (1.09-1.23; p<0.001)	1.26 (1.16-1.36; p<0.001)	2.75 (2.46-3.08; p<0.001)	1.72 (1.63-1.81; p<0.001)
Hyperhomocystinemia	138 (0.02)	0.62 (0.26-1.52; p=0.300)	0.52 (0.06-4.58; p=0.553)	3.54 (0.73-17.01; p=0.115)	1.19 (0.42-3.32; p=0.746)
Hyperuricemia	2,071 (0.3)	0.88 (0.68-1.12; p=0.300)	0.87 (0.56-1.35; p=0.537)	0.70 (0.28-1.74; p=0.444)	1.10 (0.84-1.45; p=0.494)
High leptin levels/Obesity	69,728 (10.1)	0.99 (0.95-1.04; p=0.681)	0.92 (0.85-0.99; p=0.046)	0.97 (0.84-1.12; p=0.708)	1.16 (1.10-1.21; p<0.001)
Hypertriglyceridemia	2,071 (0.3)	0.94 (0.74-1.21; p=0.634)	1.25 (0.85-1.82; p=0.255)	0.95 (0.38-2.36; p=0.918)	0.85 (0.64-1.14; p=0.283)
Anemia of CKD	382,467 (55.4)	0.96 (0.93-0.99; p=0.006)	1.05 (1.00-1.11; p=0.041)	0.56 (0.52-0.61; p<0.001)	0.93 (0.90-0.96; p<0.001)
Thrombophilia	9,665 (1.4)	1.23 (1.09-1.39; p=0.001)	1.18 (0.98-1.42; p=0.087)	5.91 (4.98-7.00; p<0.001)	1.92 (1.71-2.15; p<0.001)
Sleep disorders	34,519 (5.0)	1.13 (1.06-1.21; p<0.001)	0.933(0.84-1.04; p=0.200)	0.90 (0.74-1.10; p=0.288)	1.22 (1.14-1.30; p<0.001)
Immunosuppressive therapy	12,427 (1.8)	1.31 (1.19-1.44; p<0.001)	1.02 (0.84-1.23; p=0.862)	0.78 (0.54-1.14; p=0.207)	1.39 (1.24-1.56; p<0.001)

For acute coronary syndromes, significant predictors identified in the study included post-dialytic hypotension (p<0.001), elevated leptin levels/obesity (p=0.046), and anemia of CKD (p<0.001), while factors such as uremic syndrome, hyperhomocysteinemia, malnutrition, hyperuricemia, hypertriglyceridemia, sleep disorders, and immunosuppressive therapy were not associated with increased odds of acute coronary events in this study. Regarding sudden cardiac death, after adjusting for traditional risk factors, malnutrition, post-dialytic hypotension, anemia of CKD, and thrombophilia were found to significantly increase the odds of sudden cardiac death (p<0.001). Heart failure was the most common CV event recorded (58% of all admissions), with malnutrition (aOR: 1.07; 95% CI: 1.04-1.14; p<0.001), post-dialytic hypotension (aOR: 1.16; 95% CI: 1.09-1.23; p<0.001), thrombophilia (aOR: 1.23; 95% CI: 1.09-1.39; p=0.001), sleep disorders (aOR: 1.13; 95% CI: 1.06-1.21; p<0.001), and post-transplant immunosuppressive therapy (aOR: 1.31; 95% CI: 1.19-1.44; p<0.001) identified as significant predictors. Interestingly, anemia of CKD was associated with lower odds of heart failure (aOR: 0.96; 95% CI: 0.93-0.99; p=0.006). Uremic syndrome, hyperhomocysteinemia, obesity, and hypertriglyceridemia were not validated as significant predictors of heart failure in CKD. Regarding cardiac arrhythmias, out of 124,267 hospitalizations, 91.7% (113,912) were atrial fibrillations, 16.5% (20,711) were ventricular arrhythmias, 6.9% (8,629) were supraventricular tachycardia, and 0.1% (690) were ventricular tachycardia.

Overall, the study validated several predictors of increased CV events on multivariate analysis, including malnutrition, post-dialytic hypotension, thrombophilia, sleep disorder, and post-renal transplant immunosuppressive therapy. However, factors such as carnitine deficiency, hyperhomocysteinemia, hypertriglyceridemia, and uremic syndrome did not demonstrate significant associations with any CV events in the study (Table 2).

## Discussion

The present study analyzed a substantial number of hospitalizations (690,375) with a primary diagnosis of CKD, providing a robust dataset for investigating the relationship between CKD-specific risk factors and CV events. The sociodemographic characteristics of the study population revealed important insights. The predominance of male patients highlights the potential gender-related differences in CKD and CV risk, underscoring the need for gender-specific interventions. Furthermore, the higher proportion of admissions during weekdays suggests that the severity of CKD and the need for hospitalization may be more prevalent during working days, indicating the potential influence of occupational factors or delayed seeking of healthcare. Additionally, the majority of patients receiving care at urban teaching hospitals indicates the complexity and severity of CKD cases in these settings, highlighting the significance of specialized care and expertise required.

In line with previous investigations, a substantial proportion of CKD hospitalizations in the current study (97.5%) were covered by insurance under Medicare, Medicaid, or private insurance including HMO, while a smaller fraction represented self-pay or uninsured individuals. These findings align with a similar study conducted by Daratha et al., which aimed to identify risk factors associated with hospitalizations and mortality among CKD patients. The prevalence of public insurance coverage observed in both studies emphasizes the importance of accessible healthcare resources and insurance coverage for this vulnerable population [21].

The incidence of CV events in CKD patients addressed in this study is a crucial aspect to consider. The overall occurrence of at least one CV event in 63.2% of the hospitalizations demonstrates the substantial burden of CV disease in the CKD population. The high incidence rate of adverse CV events (158 per 1000 admissions) emphasizes the elevated risk faced by CKD patients, underscoring the urgent need for preventive strategies and improved management of risk factors to reduce CV complications in this vulnerable population.

The study further examined the prevalence of traditional and CKD-specific risk factors for CV events. Notably, a high proportion (84.1%) of hospitalizations had at least one traditional risk factor, indicating the relevance of established CV risk factors, such as hypertension, diabetes, and smoking, in CKD patients. These findings reinforce the need for comprehensive management strategies that address these traditional risk factors to mitigate the associated CV risk.

Importantly, this study investigated the impact of CKD-specific risk factors on different CV events. Individual analysis of acute coronary syndromes, cardiac arrhythmias, sudden cardiac death, and heart failure identified significant predictors for each event, providing valuable insights into the specific relationships between CKD and CV complications.

For acute coronary syndromes, the study found that post-dialytic hypotension, elevated leptin levels/obesity, and anemia of CKD were associated with increased odds of occurrence. These findings suggest that these factors may contribute to the development and progression of acute coronary syndromes in CKD patients. Consistent with a study by Okabe et al. (2023), which investigated the impact of nutritional status on the development of coronary atherosclerosis in CKD patients, the current findings highlight the notable correlation between malnutrition and the progression of coronary artery calcification [22]. However, unlike the results of the index study, a recent cohort study (Rhee et al., 2020) reported a high prevalence of uremic symptoms among CKD patients in the United States, indicating the need for further investigation into the relationship between uremic syndrome and acute coronary events [23]. Elevated homocysteine levels in CKD patients were also found to be associated with an increased risk of CV disease, underscoring the potential impact of impaired renal clearance of plasma homocysteine on CV outcomes. Additional research is necessary to better understand the roles of these factors in the development of acute coronary syndromes in CKD patients and their potential implications for clinical management.

Regarding sudden cardiac death, malnutrition, post-dialytic hypotension, anemia of CKD, and thrombophilia were identified as significant predictors of this outcome. These findings emphasize the importance of addressing nutritional status, blood pressure control, anemia management, and thrombophilia screening in CKD patients to prevent sudden cardiac death. Consistent with a related study conducted among ESRD patients undergoing hemodialysis, the duration of dialysis, hypokalemia, and specific cardiac arrhythmias such as ventricular tachycardia and ventricular fibrillation were reported as predictors of sudden cardiac death in ESRD patients [24]. Hence, it is crucial to consider these risk factors alongside traditional CV risk factors in clinical decision-making and patient management.

Heart failure emerged as the most common CV event recorded in this study. Several factors, including malnutrition, post-dialytic hypotension, thrombophilia, sleep disorders, and post-transplant immunosuppressive therapy, were identified as significant predictors of heart failure. These findings highlight the multifactorial nature of heart failure in CKD patients, with non-traditional risk factors playing a prominent role. Interestingly, anemia of CKD was associated with lower odds of heart failure, suggesting a potential protective effect. However, further investigation is warranted to elucidate the underlying mechanisms and confirm these associations. The lack of significant associations for factors such as uremic syndrome, hyperhomocysteinemia, obesity, and hypertriglyceridemia emphasizes the need for additional research to better understand their impact on heart failure in the context of CKD. Overall, these results help prioritize the management of specific risk factors and guide interventions to reduce the incidence of heart failure in CKD patients.

The study also provided insights into the specific types of cardiac arrhythmias observed in CKD patients. Atrial fibrillation accounted for the majority of cases (91.7%), followed by ventricular arrhythmias, supraventricular tachycardia, and ventricular tachycardia. These findings highlight the importance of vigilant monitoring and targeted interventions for atrial fibrillation in CKD patients due to its high prevalence. Understanding the distribution of different arrhythmias assists in tailoring treatment strategies and implementing appropriate interventions to reduce the burden of cardiac arrhythmias in this population.

Overall, the study contributes to the understanding of CKD-specific risk factors for CV events. The identification of reliable predictors allows healthcare professionals to prioritize interventions, establish preventive strategies, and tailor treatment approaches for CKD patients at higher risk of adverse CV outcomes. However, it is important to note that the study has limitations, including its retrospective design and reliance on administrative data. Future research should focus on prospective studies with detailed clinical data and diverse CKD populations to further validate and expand upon these findings.

## Conclusions

This study provides valuable insights into the CKD-specific risk factors for CV events. The analysis of a substantial number of hospitalizations reveals the high burden of CV disease in CKD patients, with a majority experiencing at least one CV event. The identification of specific risk factors associated with acute coronary syndromes, sudden cardiac death, heart failure, and cardiac arrhythmias allows for targeted interventions and tailored treatment approaches.

The study highlights the relevance of traditional CV risk factors such as hypertension, diabetes, and smoking in CKD patients, emphasizing the need for comprehensive management strategies. Additionally, it identifies CKD-specific risk factors including post-dialytic hypotension, malnutrition, anemia of CKD, thrombophilia, and obesity that significantly correlate with adverse CV outcomes. Understanding these risk factors enables healthcare professionals to prioritize interventions and implement preventive strategies to reduce the incidence of CV complications in CKD patients. Future research should focus on prospective studies with detailed clinical data to further validate and expand upon these findings. Overall, these findings have important implications for clinical decision-making and improving CV care for individuals with CKD.
